# Renal cysts in Caroli's disease

**DOI:** 10.4103/0971-4065.62089

**Published:** 2010

**Authors:** Y. Lakshmi, B. Vijaya Lakshmi Devi, S. Sarala

**Affiliations:** Departments of Radiology and Imaging, Sri Venkateswara Institute of Medical Sciences, India

A thirty-year-old male presented with vague upper abdominal pain. There were no urinary symptoms. The general and systemic examination was unremarkable. The hemoglobin was 8.8 g/dl, total leukocyte count was 11 200/cumm, blood urea was 25 mg/dl and serum creatinine was 0.9 mg/dl. The urine examination was normal. Contrast-enhanced computed tomography (CECT) of abdomen revealed dilated intra hepatic biliary ducts and presence of “central dot” sign representing intraluminal portal vein in dilated intrahepatic bile ducts in the right lobe of liver suggestive of Caroli's Disease [Figure 1]. Axial CECT showed multiple cysts in the lower poles of both kidneys [Figure 2].

**Figure 1:**
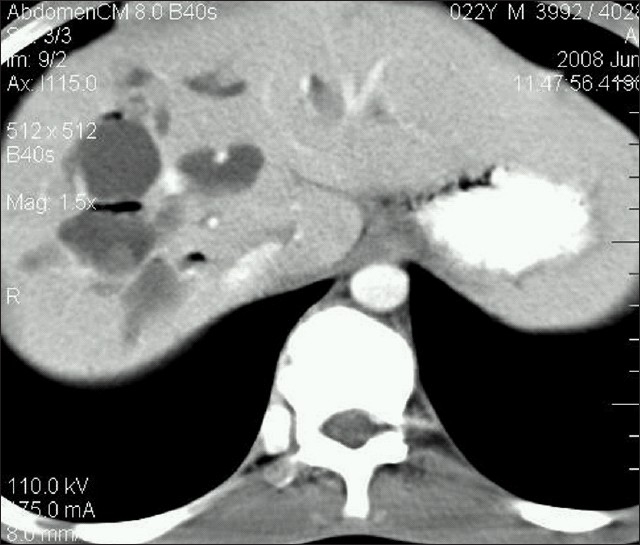
Contrast-enhanced computed tomography of abdomen showing dilated intrahepatic biliary ducts with air foci and presence of “central dot” sign representing intraluminal portal vein in dilated intrahepatic bile ducts in right lobe of liver

**Figure 2:**
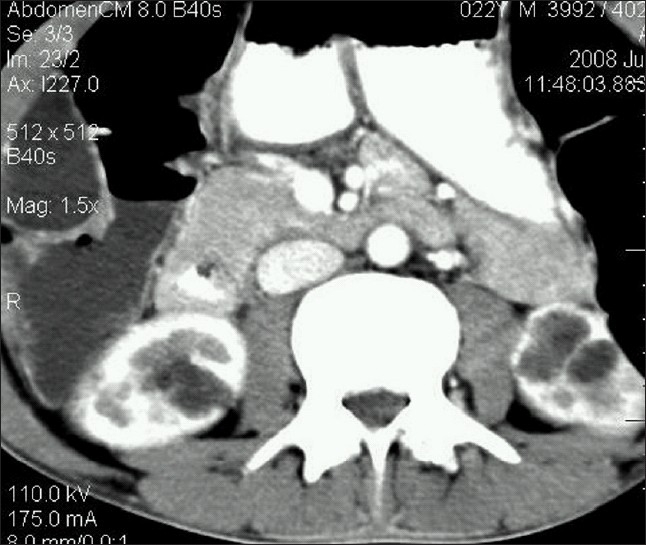
Axial contrast-enhanced computed tomography showing multiple cysts in the lower poles of both kidneys

Caroli's disease is a rare, complex autosomal recessive congenital disorder characterized by multiple focal segmental saccular dilatations of the intrahepatic bile ducts, a predisposition to biliary calculi and cholangitis and an association with various forms of cystic renal disease. The disease is common in childhood and in the second to third decades of life. Caroli's disease can be associated with varying degrees of renal cysts, renal tubular ectasia, medullary sponge kidneys, autosomal recessive kidney disease, nephrocalcinosis, interstitial fibrosis and renal failure.[1–3] To differentiate from autosomal dominant polycystic kidney disease where-in the liver and kidneys are affected, the presence of a central dot sign in CECT helps in pointing the diagnosis toward Caroli's disease. In our evaluation, we found renal cysts in both the kidneys.

## References

[1] Mrowka Ch, Adam G, Sieberth HG, Matern S (1996). Caroli's syndrome associated with medullary sponge kidney and nephrocalcinosis. Nephrol Dial Transplantation.

[2] Gore RM, Fulcher AS, Taylor AJ, Ghahremani GG (2008). Anomalies and anatomic variants of the gallbladder and biliary tract. Text book of gastrointestinal radiology.

[3] Toprak O, Uzum A, Cirit M, Esi E, Inci A, Ersoy R (2006). Oral–Facial-digital syndrome type 1, Caroli's disease and cystic renal disease. Nephrol Dial Transplant.

